# Pathogenomic Insights into *Piscirickettsia salmonis* with a Focus on Virulence Factors, Single-Nucleotide Polymorphism Identification, and Resistance Dynamics

**DOI:** 10.3390/ani15081176

**Published:** 2025-04-20

**Authors:** Sk Injamamul Islam, Khandker Shahed, Md Imtiaz Ahamed, Luu Tang Phuc Khang, Won-Kyo Jung, Papungkorn Sangsawad, Nguyen Dinh-Hung, Patima Permpoonpattana, Nguyen Vu Linh

**Affiliations:** 1 BioMac Lab, Dhaka 1217, Bangladesh; sk.islam@biomaclab.com (S.I.I.); kh.shahed@biomaclab.com (K.S.); imtiaz.24160107@bau.edu.bd (M.I.A.); 2 Department of Animal and Aquatic Sciences, Faculty of Agriculture, Chiang Mai University, Chiang Mai 50200, Thailand; khang_luu@cmu.ac.th; 3 Major of Biomedical Engineering, Division of Smart Healthcare, College of Information Technology and Convergence and New-Senior Healthcare Innovation Center (BK21 Plus), Pukyong National University, Busan 48513, Republic of Korea; wkjung@pknu.ac.kr; 4 School of Animal Technology and Innovation, Institute of Agricultural Technology, Suranaree University of Technology, Nakhon Ratchasima 30000, Thailand; papungkorn@sut.ac.th; 5 Aquaculture Pathology Laboratory, School of Animal & Comparative Biomedical Sciences, The University of Arizona, Tucson, AZ 85721, USA; dinhhung@arizona.edu; 6 Department of Agricultural Science and Technology, Faculty of Innovative Agriculture, Fisheries and Food, Prince of Songkla University, Surat Thani Campus, Surat Thani 84000, Thailand; 7 Functional Feed Innovation Center (FuncFeed), Faculty of Agriculture, Chiang Mai University, Chiang Mai 50200, Thailand

**Keywords:** genomics, pathogenesis, piscirickettsiosis, SNPs, virulence

## Abstract

Effective control of bacterial infections remains a significant challenge in aquaculture. The marine bacterium *Piscirickettsia salmonis* (*P. salmonis*), responsible for piscirickettsiosis, causes widespread infections in various salmon species, leading to substantial mortality and economic losses. Despite efforts to genetically characterize *P. salmonis*, critical gaps persist in understanding its virulence factors, antimicrobial resistance genes, and single-nucleotide polymorphisms (SNPs). This study addresses these gaps through a comparative analysis of the pan-genome and core genomes of 80 *P. salmonis* strains from different geographical regions and genogroups. *P. salmonis* had an open pan-genome consisting of 14,564 genes, with a core genome of 1257 conserved genes. Eleven virulence-related genes were identified in the pan-genome, categorized into five functional groups, providing new insights into the pathogenicity of *P. salmonis*. Unique SNPs were detected in four key genes (*gyrA*, *dnaK*, *rpoB*, and *ftsZ*), serving as robust molecular markers for distinguishing the LF and EM genogroups. Notably, AMR genes identified in four LF strains suggest evolutionary adaptations under selective pressure. Functional annotation of the core genomes using the gene ontology (GO) and Kyoto Encyclopedia of Genes and Genomes (KEGG) databases demonstrated conserved gene clusters linked to essential intracellular survival mechanisms and bacterial pathogenicity. These findings suggest a direct association between core genome features and variations in pathogenesis and host–pathogen interactions across genogroups. Phylogenetic reconstruction further highlighted the influence of AMR genes on strain divergence. Collectively, this study enhances the genomic understanding of *P. salmonis* and lays the groundwork for improved diagnostic tools and targeted therapeutics to manage piscirickettsiosis in aquaculture.

## Simple Summary

*Piscirickettsia salmonis*, a major pathogen in aquaculture, causes lethal infections in salmonids and results in substantial economic losses. This study analyzed 80 globally sourced strains to investigate the genetic diversity, virulence factors, and antimicrobial resistance (AMR) profiles of the species. The bacterium exhibited an open pan-genome of 14,564 genes and a conserved core genome comprising 1257 genes. Eleven virulence-associated genes and unique single-nucleotide polymorphisms (SNPs) in *gyrA*, *dnaK*, *rpoB*, and *ftsZ* were identified, effectively distinguishing key genogroups. The presence of AMR genes in selected strains suggests evolutionary adaptation driven by antibiotic pressure. Functional analyses linked core genes to intracellular survival and pathogenicity, shedding light on the mechanisms underlying infection variability. These findings offer critical insights for improving diagnostics, surveillance, and targeted disease management in aquaculture systems.

## 1. Introduction

Aquaculture is a rapidly expanding food sector, providing high-quality protein to the global population. In 2020, global aquaculture production reached 122.6 million metric tons, valued at USD 281.5 billion [1], helping address food shortages caused by overpopulation. Over the past five decades, production in marine and freshwater farms has grown substantially [2], with a 609% increase in annual output between 1990 and 2020, sustained by an average annual growth rate of 6.7% [3]. Salmonids, farmed extensively in Norway, Chile, the USA, the UK, and Canada [4], are key contributors to this growth. However, intensive aquaculture systems, while optimizing water use, impose significant stress on fish increasing susceptibility to bacterial diseases [5].

The marine gammaproteobacterium *Piscirickettsia salmonis (P. salmonis)*, the pathogen responsible for piscirickettsiosis, induces multi-systemic infections in salmonids, resulting in severe mortality and economic losses [6]. Clinical manifestations include ulcerative skin lesions, darkening of the skin, abdominal enlargement, pale gills (anemia) [6,7], and replication within macrophage cytoplasmic vacuoles, ultimately leading to host death [8]. Piscirickettsiosis has been reported in coho salmon (*Oncorhynchus kisutch*), Atlantic salmon (*Salmo salar*), and rainbow trout (*Oncorhynchus mykiss*) in Norway, Canada, Scotland, Ireland, and Chile [9]. In Chile alone, it accounted for 47.8% and 67.3% of transmissible disease-related mortalities in Atlantic salmon and rainbow trout, respectively, in 2020 [10]. Current control strategies include vaccination, improved husbandry practices, and, in some cases, antibiotic treatment, with ongoing research focusing on developing more effective vaccines and alternative therapies. However, their effectiveness has been limited [11,12]. Existing vaccines primarily use inactivated whole-cell bacterins; however, the complex host–pathogen interactions and intracellular survival mechanisms of *P. salmonis* have hindered the development of effective long-term treatments [9]. Recent research has focused on applying advanced technologies, such as ‘omics’ for identifying potential vaccine antigens [12] and combining MALDI-mass spectrometry with machine learning for early pathogen detection, showing promising results [13].

Genetic classification divides *P. salmonis* into two genogroups (LF-89-like and EM-90-like) [14], with Chilean EM-90 strains showing distinct genetic divergence from LF-89 isolates. Genomic and phylogenetic analyses of 16S ribosomal DNA (rDNA) confirm these groups [15], and virulence differences are evident: EM-90-like strains result in higher mortality rates and faster disease progression than LF-89 strain in Atlantic salmon [16]. Current diagnostic methods, including culture-based assays [17], conventional polymerase chain reaction (PCR) [18], real-time PCR [19], and fluorescence antibody tests [20], lack specificity for genogroup differentiation [15]. This limitation hinders effective disease management, as genogroup-specific variations in pathogenesis and immune responses influence results [21]. While PCR-restriction fragment length polymorphism (RFLP) assays targeting 16S rDNA have been developed [14], 16S rRNA sequencing alone cannot resolve species-level distinctions [22]. Although virulence genes offer potential diagnostic markers [10], few studies have explored their utility for *P. salmonis* classification. Vaccine development is further complicated by genetic variability and the absence of universal biomarkers [10]. Current bacterin, recombinant [9], and live-attenuated vaccines [12] provide incomplete protection [23] and antibiotics overuse risks AMR.

Advances in whole-genome sequencing (WGS) have greatly enhanced phylogenetic resolution, genotyping, and AMR gene detection [24,25]. A 2017 study analyzed 13 complete and 6 draft *P. salmonis* genomes to catalog virulence factors [26], but the quadrupling of available genomes since necessitates updated genomic insights. Horizontal gene transfer—not vertical inheritance—drives virulence and AMR gene acquisition in *P. salmonis* [27], complicating taxonomic relationships. This study employs comparative genomics of 80 *P. salmonis* genomes to construct pan and core genomes, calculate average nucleotide identity (ANI), and assess genetic diversity. By identifying novel single-nucleotide polymorphisms (SNPs) and virulence determinants, we provide critical insights into strain pathogenicity and propose robust molecular markers for precise genogroup differentiation.

## 2. Materials and Methods

### 2.1. Genomic Dataset Selection

A total of 80 complete genomic sequences of *P. salmonis* were retrieved from the National Center for Biotechnology Information (NCBI) in March 2024. Inclusion criteria required genome completeness and taxonomic identification as *P. salmonis*. Additionally, the metadata and BioSample records for each genome were collated (Supplementary Table S1). Only whole-genome sequences were analyzed to ensure accurate characterization of virulence genes and biomarker identification.

### 2.2. Species Validation Using Average Nucleotide Identity

To validate species relationships, we performed nucleotide-level comparisons of all possible genome combinations using the ANI method. The Python script PyANI v0.2.722 was used to compute pairwise ANI values using two different methods, the MUMmer [28] and the BLAST (https://www.ncbi.nlm.nih.gov, accessed on 2 July 2024) method [29] employing the ANIm and ANIb options, respectively. All strains identified as *P. salmonis* were selected for further investigation, while those incorrectly identified were excluded. This was determined by establishing a threshold value indicating that the presence of the same species of ANI was greater than 94% [30,31].

### 2.3. Pan-Genome Analysis

Genomes were uniformly re-annotated for their functionality to ensure consistency and standardization using Prokka v1.14.6 [32]. Pan-genome and core genome were constructed with Roary v3.13.0 [33] at a 95% identity threshold. Genes were categorized into four distinct groups, including core genes present in ≥99% of strains; soft-core which was of 95–99% of strains; shell which was of 15–95% of strains; and cloud genes which were of <15% of strains. Additionally, pan-genome was analyzed using Heap’s Law [34] and a Power Law fit [35], along with a custom script (https://github.com/SethCommichaux/Heap_Law_for_Roary, access on 2 July 2024) and the “ggcaller v1.3.0” in Python programming [36], respectively. This approach enabled the calculation of constant variables and the application of the least squares method to fit an exponential regression decay model to both the core genome and singletons. Heap’s Law was utilized to determine the fixed parameters from the pan-genome with the formula *n* = κN^γ^, where *n* represents the number of pan-genome genes and N^γ^ is the number of genomes. If γ < 0, the pan-genome was considered closed; if γ > 0, it was open. Least-squares fit was represented by the equation *n* = *k* × exp [−x/*t*] + tgθ, where *n* is the number of genes, and *k*, *t*, and tgθ are independent variables. This algorithm was employed to determine the number of genes that composed the core genome upon stabilization [37] and to provide an approximate calculation of the genes contributed by each freshly sequenced genome [25].

### 2.4. Phylogenetic Analysis and SNP Identification

Evolutionary connections and unique genes were determined using the genes *dnaK, gyrA, rpoB*, and *ftsZ*, whose sequences were retrieved during pan-genome analysis [26]. An appropriate evolutionary model was selected after acquiring numerous sequence alignments using MUSCLE with default parameters (Gap Open Penalties = −400.00/Gap Extend Penalties = 0.00). Subsequently, Fast Tree v2.10 was used to reconstruct the phylogenetic tree with the most significant likelihood approach and appropriate substitution model. All positions with less than 95% coverage were eliminated. A bootstrap value of 500 was assigned. The CLUSTALW format for multiple sequence alignment was generated using T-Coffee methods to detect significant SNPs that were used for genotyping or molecular identification. A customized Python script msa2snp.py (available at https://github.com/pinbo/msa2snp, access on 2 July 2024) was employed to detect all SNPs within the target genes. SNP identification was manually curated to identify the most informative SNPs found exclusively in all *P. salmonis* genomes.

### 2.5. Functional Annotation and Identification of Gene Encoders

BLAST searches were performed for all protein-coding genes in the NCBI database, which was accessed on 2 July 2024. The entire genome, including repetitive elements, was annotated using eggNOG-mapper v.2.0 [38] in Linux programming. The sequence alignment process entailed mapping each sequence with either the hidden Markov model (HMM) or DIAMOND to match it with the eggNOG database. The ideal matching sequence of the target sequence was classified according to its taxonomy and further categorized and annotated utilizing GO [39] and KEGG pathways [40] through the application of the cluster Profiler v.3.19 package in R [41]. Furthermore, BLAST analysis was performed to examine the 80 genomes for the presence of genes linked to virulence and pathogenicity. We employed BLASTN via ABRicate v1.0.1 (https://github.com/tseemann/abricate, access on 2 July 2024) [42] and the Virulence Factor Database (VFDB) [43] to examine the occurrence of virulence factors. The analysis was performed using a 90% identity threshold and a 30% coverage threshold.

## 3. Results

### 3.1. Genomic Features and ANI

**Figure 1:**
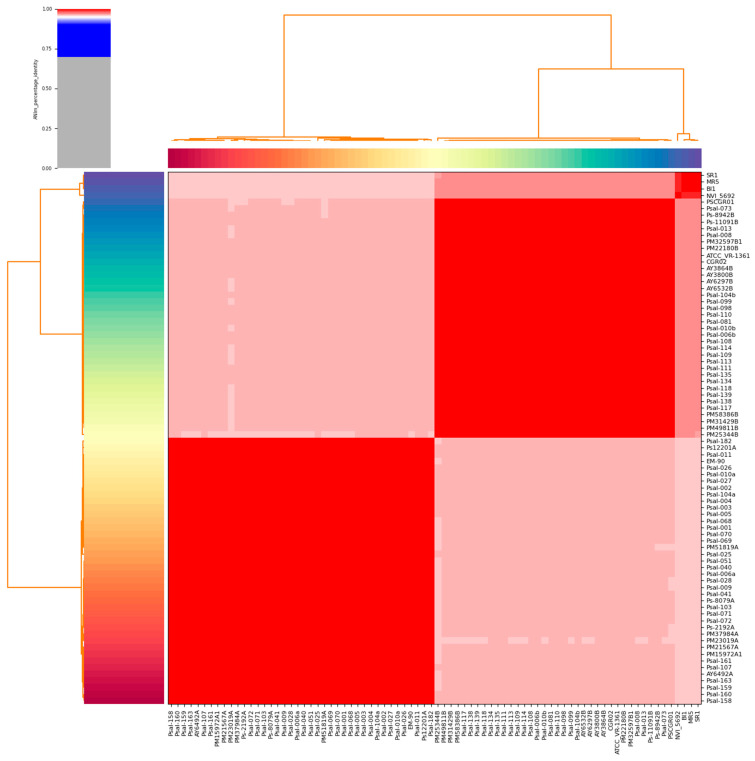
Heat map displays the pairwise average nucleotide identity (ANI) values for 80 genomes of *P*. *salmonis*. The utilization of color coding on the *x-axis* and *y-axis* was employed to distinguish between the various strains of genomes. The red color indicates a significant level of resemblance, implying that the organisms belong to the same species.

The overall genomic characteristics of the *P. salmonis* strains used in this investigation are shown in Supplementary Table S1. The genomic analysis of *P. salmonis* revealed significant variability across 80 sequenced genomes, providing insights into the genetic functions of pathogenic bacteria. The genome sizes ranged from 3.24 Mb to 4.15 Mb. Interestingly, larger genomes, such as those of strains MR5 (4.14 Mb) and SR1 (4.15 Mb), exhibited a higher number of chromosomes compared to smaller genomes such as strain PM15972A1 (3.24 Mb). The gene content in these genomes also correlated with their size, with larger genomes containing more genes. For instance, strain SR1, with the largest genome, had 4955 genes, while smaller genomes, such as strain PM15972A1, had fewer (3365 genes). The genome assemblies exhibited several chromosomes ranging from 2 to 13, with most strains containing 4–6 chromosomes. ANI analyses showed that the strains were classified into three major clades (Figure 1), with one of them containing the highest number of strains. The ANI analysis of 80 *P. salmonis* strains revealed a highly homogeneous genomic landscape, with ANI values exceeding 95%, underscoring their close genetic relatedness. Within this dataset, distinct clades emerged—Clade I (e.g., strains MR5 and SR1) exhibited extremely high genetic similarity (ANI: 98.5–99.8%); Clade II (e.g., strains Psal-009, Psal-010b, and NVI 5692) showed moderate variability (ANI: 97.8–99.5%); and Clade III (e.g., strains ATCC VR-1361 and Psal-070) exhibited a broader spectrum of ANI values, typically exceeding 97.5%.

### 3.2. Pan-Genome Characteristics

**Figure 2:**
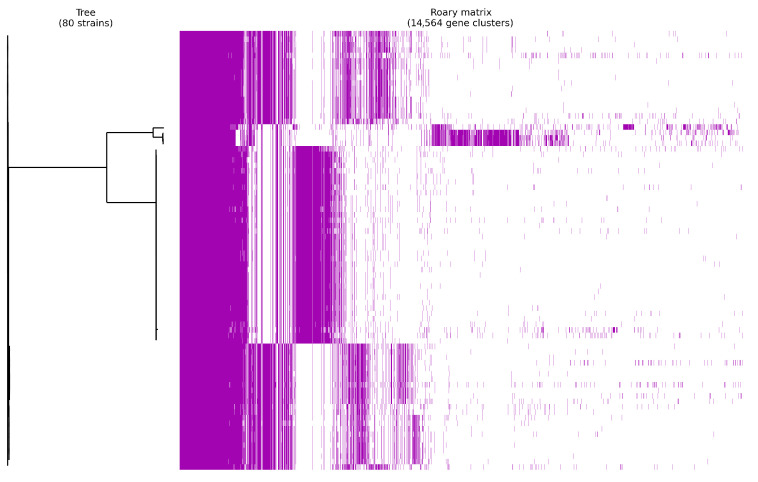
Visualization of the pan-genome of *P. salmonis* was performed using Roary v3.11.2, including 80 true genomes. The genomes of the strains were grouped based on the presence or absence of genes. The blue color indicates the presence of genes, while the color white indicates the absence of genes.

Pan-genome analysis was conducted to assess virulence gene variability across *P. salmonis* strains (Figure 2). The analysis included 80 strains, with gene presence/absence patterns used to construct the pan-genome. The total pan-genome size was projected to contain 14,564 genes, categorized into four groups: core (79–80 genomes), soft-core (76–79 genomes), shell (12–76 genomes), and cloud (1–12 genomes) (Supplementary Figure S1). Cloud genes dominated the pan-genome (8900 genes), suggesting widespread conservation across the *P. salmonis* population. Supplementary Figure S2 illustrates the frequency distribution of gene presence, confirming core genes (present in all/nearly all genomes) and accessory genes (subset-specific), highlighting a diverse accessory genome with numerous strain-specific genes. Supplementary Figure S3 further demonstrated that most gene clusters contain few genes, though a subset exhibits large clusters (about 80 genes per cluster).

**Figure 3:**
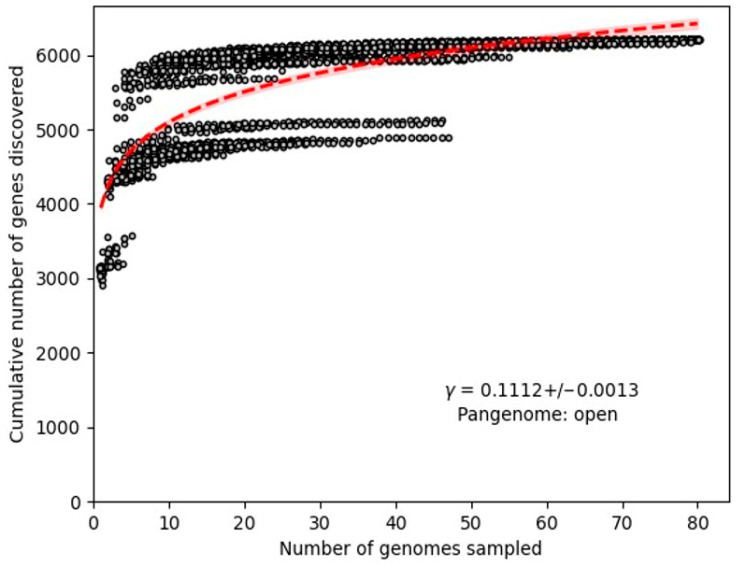
A rarefaction curve illustrates the total number of novel genes identified by the random incorporation of a single genome. The equation of the power-law fit is γ = 0.1112+/−0.0013.

The rarefaction curve shows a steady increase in the number of genes discovered with the addition of new genomes (Figure 3), indicating an open pan-genome. The power-law fit confirmed this openness, as the curve did not plateau. The relationship followed a power law pattern, evident in the approximately linear correlation between the logarithm of the cumulative gene count and the logarithm of the number of genomes sampled. The slope of the linear regression fit on a log–log scale, represented by γ = 0.1112+/−0.0013, corresponds to the power law exponent governing gene discovery [35]. With the power-law framework, the exponent γ reflects the rate of novel genes identification. A lower γ value indicated a slower discovery rate, necessitating extensive genome sampling to uncover new genes. Conversely, a higher γ would indicate rapid gene discovery with fewer genomes. The measure γ = 0.1112 suggests a large pan-genome, requiring substantial genomic data to capture its full diversity. Furthermore, the study of the pan-genome demonstrated that the γ value derived from Heap’s Law, when considering all genomes, was significantly greater than 1, showing the openness of the pan-genome.

### 3.3. Phylogenetic Relationship and SNP Identification

**Figure 4:**
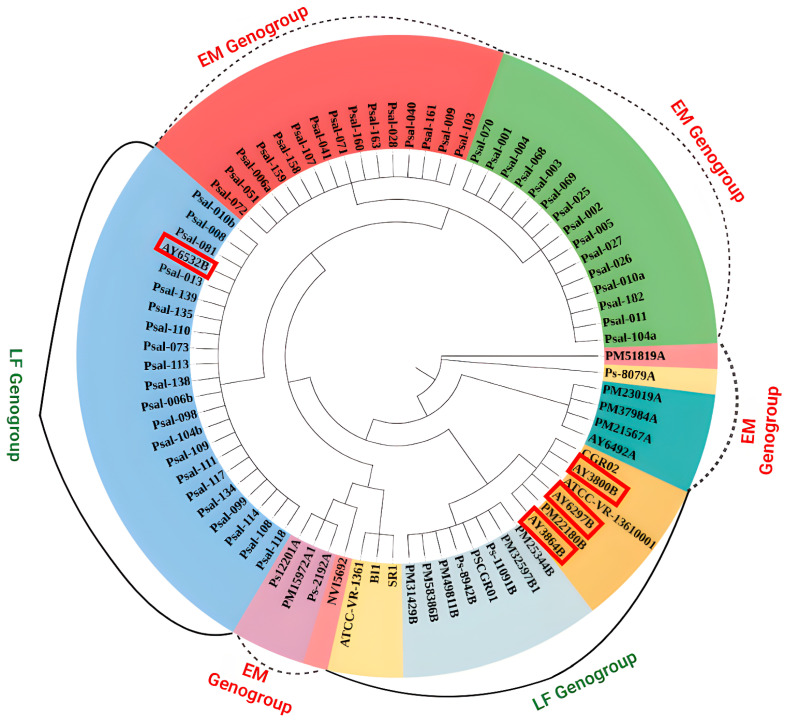
A reconstructed phylogenetic tree was generated using the *gyrA* gene and a total of 80 samples. The tree was constructed using maximum likelihood and the Kimura 2-parameter model, which was selected as the most parsimonious model. All positions with less than 95% coverage were removed. The color coding indicates different clades and the genetic variation between the strains. The red box in the LF genogroup indicates the strains which found positive for several antimicrobial resistance genes.

Phylogenetic analysis was conducted to assess the potential of the four notable genes (*dnaK*, *ftsZ*, *gyrA*, and *rpoB*) to differentiate *P. salmonis* strains (Figure 4). Through comprehensive pan-genome analysis, SNPs were identified in these genes across multiple *P. salmonis* strains. These genes were selected as candidate molecular markers for strain distinguishment based on their variability. The phylogenetic tree, constructed from the combined sequences of these genes, displayed clades consistent with the genomic variation observed in the *P. salmonis* population. Notably, *gyrA* harbored a substantial number of SNPs, demonstrating strong discriminatory power. The phylogenetic tree precisely matched the categorization derived from the ANI analysis, which was based on the comprehensive investigation of the entire genome. Therefore, this study suggests these four genes as effective diagnostic markers for strain differentiation, comparable to the ANI-based classification. For example, the *gyrA* phylogeny exhibited perfect concordance with ANI groupings, resolving distinct genogroups (LF and EM). These genogroups reflect genetic diversity and evolutionary divergence within *P. salmonis*, providing insights into genomic variability and epidemiological relevance across strains.

Furthermore, Table 1 presents the proposed gene names and their corresponding functional annotations. This table compiles the distinct SNPs observed in each gene, which can be utilized to differentiate between various strains of *P. salmonis*. The SNP records indicated that *gyrA* had the highest number of unique SNPs (64), whereas *rpoB* exhibited only two distinct SNPs.

### 3.4. Functional Annotation and Gene Encoding Factor Identification

To identify virulence genes in *P. salmonis*, the Roary output was screened and compared with VFDB-indexed genes. The identified genes are presented in Table 2, along with their functional categories and annotated product names. Notably, most strains possessed virulence genes with >83% sequence similarity, with few exceptions. For example, the *feoB* gene was detected in only 17 of the 80 analyzed strains, while *icmO/dotL*, *ggt, htpB, tufa*, and *rposA* genes were found in all the strains. The pan-genome analysis of *P. salmonis* against the VFDB database revealed a variety of virulence-associated genes distributed across different functional categories. These genes involved in nutritional/metabolic factors like *ggt* (gamma-glutamyltranspeptidase) and *feoB* (ferrous iron transporter B), adherence factors such as *htpB* (Hsp60) and *tufA* (elongation factor Tu), along with regulatory genes like *csrA* and *rpoS* (RNA polymerase sigma factor). Additionally, the analysis identified genes linked to motility, including *flrA*, *fliA*, and *flhA*, and an effector delivery system gene, *icmO/dotL*, associated with the Dot/Icm type IV secretion system. Additionally, antibiotic-resistance genes, including chloramphenicol, streptomycin, and tetracycline were identified in four strains such as AY3800B, AY6297B, AY6532B, and AY3864B.

**Figure 5:**
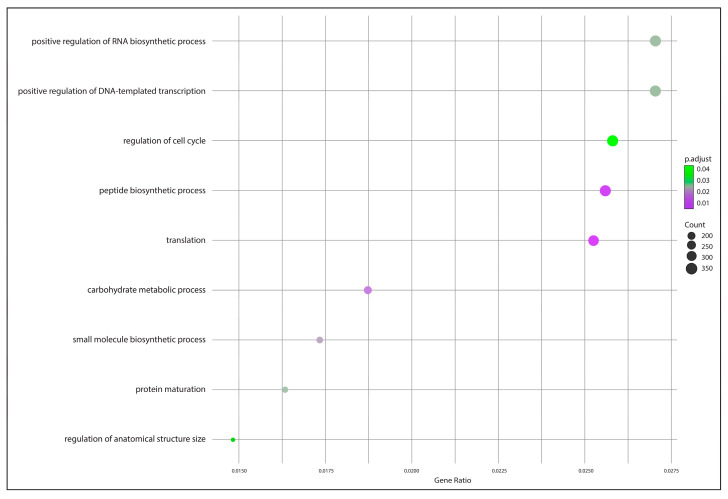
GO enrichment analysis of core genes from the *P. salmonis* pan-genome using zebrafish as a reference model.

The GO enrichment analysis depicted in Figure 5 highlights several biological processes associated with core genes identified from the *P. salmonis* pan-genome using zebrafish as the reference model. Significant enrichment was observed in processes such as positive regulation of RNA biosynthetic process, positive regulation of DNA-templated transcription, and regulation of the cell cycle. Other notable processes included peptide biosynthetic process, translation, and carbohydrate metabolic process.

**Figure 6:**
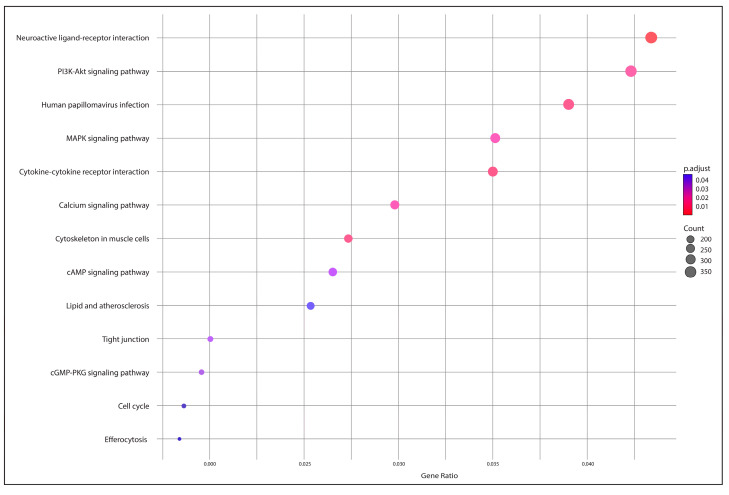
KEGG pathway enrichment analysis of core genes from the *P. salmonis* pan-genome.

Additionally, Figure 6 presents the KEGG pathway enrichment analysis of core genes identified from the pan-genome analysis. The result revealed that the neuroactive ligand–receptor interaction pathway had the highest number of associated genes, while pathways like efferocytosis and the cell cycle had the fewest.

## 4. Discussion

The *P. salmonis* genome comprises multiple genotypes and strains, making it challenging to assess virulence through phenotypic characterization alone [14]. While taxonomic classification at the genomic level can identify strains with potential differences in virulence, a comprehensive evaluation requires infection models to examine host–pathogen interactions. This is critical because genes associated with virulence may serve alternative functions in different hosts, limiting the reliability of genomic data alone. As a pathogen ubiquitous in aquatic environment, *P. salmonis* causes significant diseases in *Salmo* species [10]. Comparative genomics and in silico approaches [44] facilitate the discovery of biomarkers and molecular markers for epidemiologically important microorganisms [45]. While these methods provide insights into evolutionary relationships and molecular mechanisms, limitations remain, including variability in genome sequence quality, inconsistencies in annotation across tools, and challenges in interpreting genetic variation amid evolutionary divergence [31].

Identifying this pathogen is difficult due to its genetic variability and its close association with multiple strains. Despite the long-standing discovery of *P. salmonis,* our understanding of its pathogenicity and virulence pathways remains incomplete [46]. Misidentification of *P. salmonis* strains can result in inaccurate diagnoses, inappropriate treatments, and increased fish mortality. The outcomes contribute to greater morbidity and mortality, heightened antibiotic resistance, increased healthcare costs, and a higher likelihood of disease outbreaks. It is therefore essential to employ reliable and accurate methods such as molecular techniques, biochemical analyses, and serological tests to detect and classify the pathogen’s virulence groups.

Several studies utilizing phylogenetic techniques based on 16S rRNA sequences have identified two distinct genogroups within *P. salmonis*, corresponding to strains LF-89 and EM-90 [15]. These genogroups differ in geographic distribution, antibiotic susceptibility, and host specificity [47]. In a previous study, phylogenetic analysis was performed in thirteen complete and six draft genomes of *P. salmonis* using seven conserved single-copy genes (*dnaK*, *groEL*, *recA*, *gyrA*, *gyrB*, *rpoB*, and *ftsZ*) [26]. However, the number of *P. salmonis* genomes in public databases has since been increased fourfold. At the time of the previous study, most available genomes were still in the draft stage, limiting phylogenomic reconstruction due to an inability to identify SNPs in these single-copy genes. In the current study, no SNPs were observed in the *groEL*, *gyrB*, or *recA* genes, highlighting the limitation of earlier analyses. We propose four other single-copy genes, including *dnaK*, *gyrA*, *rpoB*, and *ftsZ*, as novel markers to differentiate pathogenic *P. salmonis* strains. These genes may serve as a viable method genotyping approach based on strain-specific genetic variation. Furthermore, the LF genogroup (AY3800B, AY6297B, AY6532B, and AY3864B) was found to contain AMR genes, as shown in Figure 4, suggesting that these strains may be undergoing evolutionary changes due to selective pressures, with identifiable SNPs characterizing the group [48]. Constructing a single-copy core phylogenomic tree and identifying AMR-associated strains within the LF group enhances our understanding of *P. salmonis* evolution, informs the spread of resistance, and supports the development of targeted diagnostics, treatment strategies, and vaccines [9].

Phylogenetic analysis of these genes revealed a consistent pattern with ANI, supporting a gene-level approach to *P. salmonis* differentiation over more complex methods, such as those proposed by Beaz-Hidalgo et al. [49]. Using a single, well-selected gene for microbiological typing offers advantages in speed, cost-effectiveness, and diagnostic efficacy. SNP-based methods have also been proposed for accurately identifying specific *Bacillus* species responsible for foodborne illness [50]. Likewise, the SNPs identified in this study provide a strong foundation for developing a novel molecular typing method capable of precisely identifying *P. salmonis* strains.

The status of a species’ pan-genome as “open” or “closed” depends on its capacity to acquire exogenous DNA [51], a trait frequently observed in bacterial species that inhabit microbial communities. These organisms typically possess large genomes, exhibit high rates of horizontal gene transfer and contain multiple ribosomal operons [52]. The results of this study suggest that the *P. salmonis* pan-genome is currently in an open state. To approach closure of the pan-genome, it is crucial to prioritize the sequencing of complete genomes from diverse isolates. The increase in core proteins observed in this study likely reflects the use of more comprehensive genome assemblies compared to those employed in earlier studies [16].

Pathogenomics, a branch of science that employs genomic tools to microbial pathogenicity, is often limited by the small number of genes analyzed in most epidemiological studies [53,54]. In this study, eleven virulence genes were identified within the *P. salmonis* pan-genome, each showing over 80% sequence identity and 90% coverage in BLAST searches. The presence of these genes contributes to the complexity of this pathogen and underscores the necessity of precise identification. Gene annotation revealed functional similarities and differences between strains and genogroups, based on KEGG metabolic pathways and gene ontology categories. Consistent with prior research on Gammaproteobacteria [55], nucleotide, lipid, translation, and carbohydrate metabolic pathways were found to be well conserved across *P. salmonis* genogroups.

Genomic analysis of *P. salmonis* in this study highlights the potential to overcome current constrains in the management of piscirickettsiosis. The identification of novel virulence factors suggests potential drug targets for future inhibitor development, which could be validated via high-throughput screening. Rotating antibiotics to mitigate resistance is also supported by resistance mechanisms and *gyrA* mutations identified in this current research. Furthermore, the SNP profiling provides a framework for multiplex PCR-based diagnostic tools, which further improves strain detection. Taken together, these strategies provide actionable paths forward, addressing treatment and diagnostic challenges in aquaculture.

## 5. Conclusions

This study provides an updated comparative analysis of *P. salmonis* genomics through pan-virulence gene identification and pathogenic potential evaluation. Four genes (*dnaK*, *gyrA*, *rpoB*, and *ftsZ*) emerged as key molecular markers for genogroup differentiation. The coexistence of AMR genes and group-defining SNPs in the LF genogroup evolutionary adaptation, potentially challenging current phylogenetic models if HGT or adaptive evolution occur. These findings enhance understanding of *P. salmonis* pathogenicity, emphasizing genes regulating virulence expression protein synthesis, and degradation that is critical for infection establishment and bacterial proliferation. This study informs targeted diagnostics, therapies, and vaccine development, addressing urgent needs in aquaculture disease management.

## Figures and Tables

**Table 1:** Differential genes and their characteristic SNPs in the *P. salmonis* genomes.

Genes	Annotation	Position, SNPs Unique for *P. salmonis*
*dnaK*	Chaperone protein	5 C > T/A, 408 A > T/-/G, 717 A > T/C, 724 G > T/A, 807 A > T/-/C, 1325 G > T/A, 1327 G > A/C, 1445 T > G/C, 1452 T > G/C, 1546 A > T/G, 1555 G > A/C, 1560 T > A/G, 1565 C > T/A, 1596 T > -/G/C, 1608 C > T/A/G, 1617 G > A/C, 1627 C > A/G, 1630 G > T/A,1690 A > T/G, 1697 A > T/G, 1717 G > A/C, 1761 T > -/A/G,
*ftsZ*	Filamenting temperature-sensitive mutant Z	297 T > G/C, 423 T > A/C, 459 A > G/T, 537 A > T/C, 612 C > CA/CG, 633 T > A/G, 634 G > T/C, 864 T > A/G, 876 C > G/T, 906 A > G/T, 975 T > A/C, 993 G > T/C, 1050 G > T/C, 1077 G > A/T, 1116 G > T/C,
*gyrA*	DNA gyrase A	36 A > /AT, 57 G > C/A/-, 192 T > G/C/-, 216 C > G/A/-, 373 A > G/C, 376 G > T/C, 380 T > G/C, 381 A > T/C, 382 T > G/C, 388 C > G/T, 390 G > T/C, 392 T > C/A, 397 C > G/T, 399 A > T/C, 402 G > T/C, 404 T > G/A, 406 T > C/A, 416 G > T/C, 419 A > G/C, 422 T > C/A, 428 A > G/C, 429 A > T/C, 431 A > G/T, 432 A > G/T, 433 A > T/C, 437 A > G/C, 440 T > G/C, 441 G > C/A, 443 T > C/A, 459 A > T/C, 461 C > G/T, 463 C > T/A, 468 T > G/A, 479 A > G/C, 480 G > C/A, 486 A > G/T, 493 G > T/A, 494 G > C/A, 495 A > G/T, 498 G > T/C, 501 G > C/A, 502 A > G/C, 505 C > T/A, 514 C > G/A/CAA, 518 G > T/A, 2106 A > G/T/-, 2343 C > G/A/-,
*rpoB*	RNA polymerase, beta subunit	1887 G > T/C, 2655 G > A/C,

**Table 2:** Functional category of 11 virulence genes in *P. salmonis* pan-genome.

Gene Functional Category	Virulence Gene	Product	Replicon
Nutritional/Metabolic factor	*ggt*	gamma-glutamyltranspeptidase	Chromosome
Adherence	*htpB*	Hsp60, 60K heat shock protein	Chromosome
Adherence	*tufA*	Elongation factor Tu	Chromosome
Effector delivery system	*icmO/dotL*	Dot/Icm type IV secretion system coupling protein	Chromosome
Motility	*flrA*	sigma-54 dependent transcriptional activator	Chromosome I
Adherence	*rpoS*	RNA polymerase sigma factor	Chromosome
Regulation	*csrA*	carbon storage regulator	Chromosome
Regulation	*rpoS*	RNA polymerase sigma factor	Chromosome
Motility	*fliA*	flagellar biosynthesis sigma factor	Chromosome I
Motility	*flhA*	flagellar biosynthesis protein	Chromosome I
Nutritional/Metabolic factor	*feoB*	ferrous iron transporter B	Chromosome

## Data Availability

The original contributions presented in this study are included in the article. Further inquiries can be directed to the corresponding author(s).

## Supplementary Materials

The following supporting information can be downloaded at: https://www.mdpi.com/article/10.3390/ani15081176/s1, Figure S1: Pangenome distribution pie chart generated by Roary analysis from 80 genomes of *P. salmonis*; Figure S2: Pangenome frequency analysis among all the 80 genomes of *P. salmonis*; Figure S3: A frequency distribution of clusters by proportion of all the strains of *P. salmonis*; Table S1: The overall statistics of all the 80 genomes of *P. salmonis* strains.
