# Supplementary material for: Pathogenomic Insights into Piscirickettsia salmonis with a Focus on Virulence Factors, Single-Nucleotide Polymorphism Identification, and Resistance Dynamics
Source: Animals (Basel). 2025 Apr 20;15(8):1176. doi: 10.3390/ani15081176 (PMC12024244; doi:10.3390/ani15081176)

animals

Supplementary Figures

Pathogenomic Insights into *Piscirickettsia salmonis* with a Focus on Virulence Factors, SNP Identification, and Resistance Dynamics

Figure S1. Pangenome distribution pie chart generated by Roary analysis from 80 genomes of *P. salmonis*.

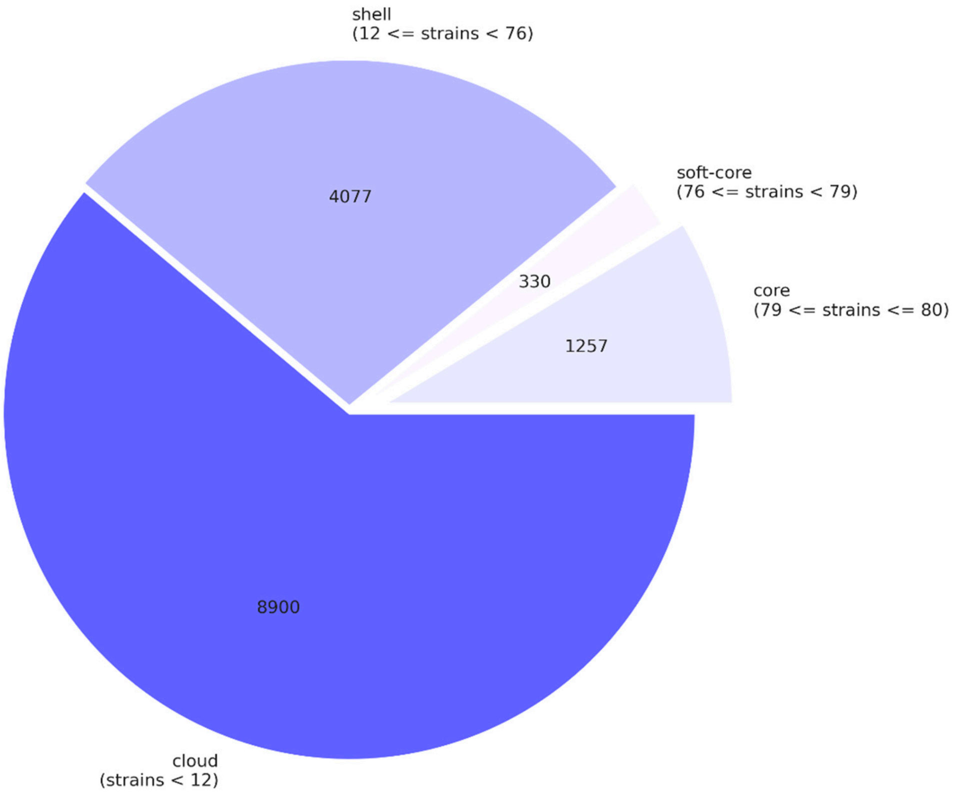

 https://doi.org/10.3390/ani15081176

Figure S2. Pangenome frequency analysis among all the 80 genomes of *P. salmonis*.

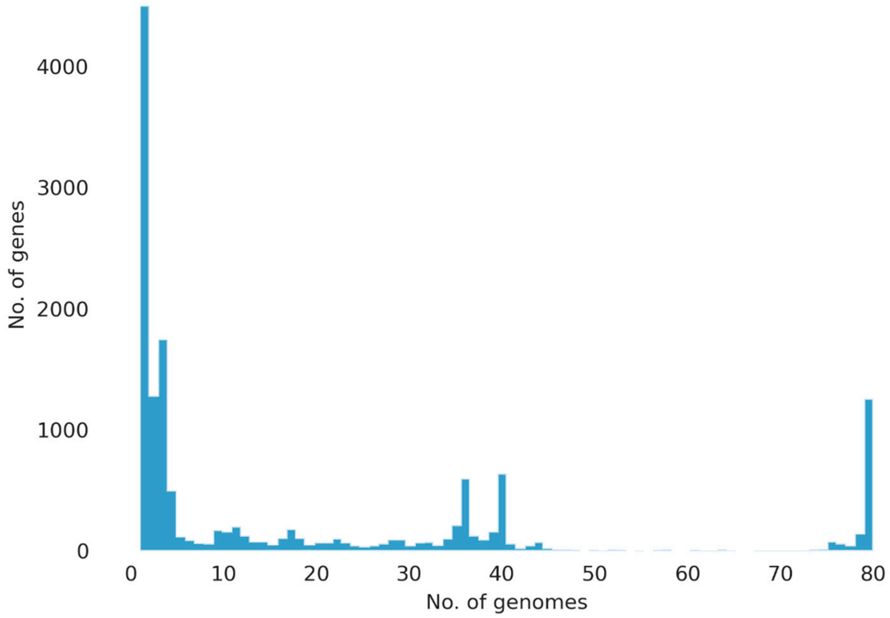

Figure S3. A frequency distribution of clusters by proportion of all the strains of *P. salmonis*

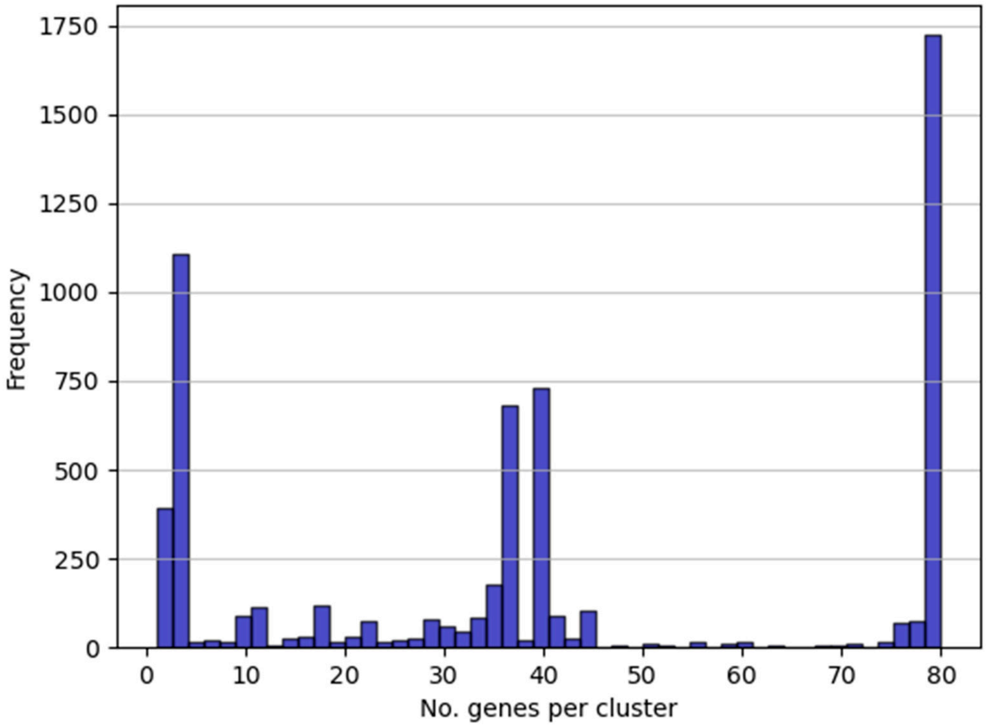

Supplement: Supplementary file 1 [file animals-15-01176-s001.zip › Supplementary Figures.pdf]
