# Supplementary material for: Pathogenomic Insights into Piscirickettsia salmonis with a Focus on Virulence Factors, Single-Nucleotide Polymorphism Identification, and Resistance Dynamics
Source: Animals (Basel). 2025 Apr 20;15(8):1176. doi: 10.3390/ani15081176 (PMC12024244; doi:10.3390/ani15081176)
Supplement: Supplementary file 1 [file animals-15-01176-s001.zip › Supplementary Tables.pdf]

animals

MDPI

Supplementary Materials

Pathogenomic Insights into Piscirickettsia salmonis with a Focus on Virulence Factors, SNP Identification, and Resistance Dynamics

Table S1. The overall statistics of all the 80 genomes of *P. salmonis* strains

Assembly Accession	*Organism Name*	Organism Infraspecific Names Strain	Annotation Name	Assembly Stats Total Sequence Length	Assembly Stats Total Number of Chromosomes	Assembly Level	Assembly Release Date	Assembly Sequencing Tech	Assembly Submitter	Annotation Count Gene Total
GCA_009708955.1	*Piscirickettsia salmonis*	Psal-070	Annotation submitted by Leibniz Institute DSMZ	3351503	3	Complete Genome	11/26/2019 0:00	PacBio; Illumina	Leibniz Institute DSMZ	3569
GCA_000300295.4	*Piscirickettsia salmonis*	ATCC VR-1361	NCBI Prokaryotic Genome Annotation Pipeline (PGAP)	3507509	5	Complete Genome	12/17/2015 0:00	Illumina GAIIx; PacBio RSII	Macrogen	3524
GCA_009709535.1	*Piscirickettsia salmonis*	MR5	Annotation submitted by Leibniz Institute DSMZ	4145588	7	Complete Genome	11/26/2019 0:00	PacBio; Illumina	Leibniz Institute DSMZ	4927
GCA_009724905.1	*Piscirickettsia salmonis*	SR1	Annotation submitted by Leibniz Institute DSMZ	4149824	7	Complete Genome	11/26/2019 0:00	PacBio; Illumina	Leibniz Institute DSMZ	4955
GCA_009709515.1	*Piscirickettsia salmonis*	BI1	Annotation submitted by Leibniz Institute DSMZ	4070014	6	Complete Genome	11/26/2019 0:00	PacBio; Illumina	Leibniz Institute DSMZ	4827
GCA_009708435.1	*Piscirickettsia salmonis*	Psal-009	Annotation submitted by Leibniz Institute DSMZ	3557522	5	Complete Genome	11/26/2019 0:00	PacBio; Illumina	Leibniz Institute DSMZ	3826
GCA_009709495.1	*Piscirickettsia salmonis*	NVI 5692	Annotation submitted by Leibniz Institute DSMZ	3764915	8	Complete Genome	11/26/2019 0:00	PacBio; Illumina	Leibniz Institute DSMZ	4125
GCA_012272975.1	*Piscirickettsia salmonis*	Ps-2192A	NCBI Prokaryotic Genome Annotation Pipeline (PGAP)	3701548	10	Complete Genome	4/12/2020 0:00	Illumina MiSeq + Oxford Nanopore MinIon	CMM	3977
GCA_009708755.1	*Piscirickettsia salmonis*	Psal-013	Annotation submitted by Leibniz Institute DSMZ	3492757	5	Complete Genome	11/26/2019 0:00	PacBio; Illumina	Leibniz Institute DSMZ	3594

GCA_010092565.1	*Piscirickettsia salmonis*	Ps-8942B	NCBI Prokaryotic Genome Annotation Pipeline (PGAP)	3713500	10	Complete Genome	2/1/2020 0:00	PacBio Sequel	CMM	3895
GCA_001534725.1	*Piscirickettsia salmonis*	CGR02	NCBI Prokaryotic Genome Annotation Pipeline (PGAP)	3423535	4	Complete Genome	1/25/2016 0:00	Illumina; PacBio	University of Chile	3435
GCA_009708575.1	*Piscirickettsia salmonis*	Psal-008	Annotation submitted by Leibniz Institute DSMZ	3453879	4	Complete Genome	11/26/2019 0:00	PacBio; Illumina	Leibniz Institute DSMZ	3563
GCA_009708355.1	*Piscirickettsia salmonis*	Psal-006b	Annotation submitted by Leibniz Institute DSMZ	3602544	6	Complete Genome	11/26/2019 0:00	PacBio; Illumina	Leibniz Institute DSMZ	3696
GCA_009709055.1	*Piscirickettsia salmonis*	Psal-099	Annotation submitted by Leibniz Institute DSMZ	3513411	5	Complete Genome	11/26/2019 0:00	PacBio; Illumina	Leibniz Institute DSMZ	3593
GCA_009708715.1	*Piscirickettsia salmonis*	Psal-010b	Annotation submitted by Leibniz Institute DSMZ	3512644	5	Complete Genome	11/26/2019 0:00	PacBio; Illumina	Leibniz Institute DSMZ	3555
GCA_009709035.1	*Piscirickettsia salmonis*	Psal-098	Annotation submitted by Leibniz Institute DSMZ	3512453	5	Complete Genome	11/26/2019 0:00	PacBio; Illumina	Leibniz Institute DSMZ	3588
GCA_009709255.1	*Piscirickettsia salmonis*	Psal-135	Annotation submitted by Leibniz Institute DSMZ	3522846	5	Complete Genome	11/26/2019 0:00	PacBio; Illumina	Leibniz Institute DSMZ	3608
GCA_009709155.1	*Piscirickettsia salmonis*	Psal-111	Annotation submitted by Leibniz Institute DSMZ	3514709	5	Complete Genome	11/26/2019 0:00	PacBio; Illumina	Leibniz Institute DSMZ	3596
GCA_009709235.1	*Piscirickettsia salmonis*	Psal-134	Annotation submitted by Leibniz Institute DSMZ	3522820	5	Complete Genome	11/26/2019 0:00	PacBio; Illumina	Leibniz Institute DSMZ	3623
GCA_009709195.1	*Piscirickettsia salmonis*	Psal-117	Annotation submitted by Leibniz Institute DSMZ	3504177	5	Complete Genome	11/26/2019 0:00	PacBio; Illumina	Leibniz Institute DSMZ	3588
GCA_009709275.1	*Piscirickettsia salmonis*	Psal-138	Annotation submitted by Leibniz Institute DSMZ	3512145	5	Complete Genome	11/26/2019 0:00	PacBio; Illumina	Leibniz Institute DSMZ	3594
GCA_009724885.1	*Piscirickettsia salmonis*	Psal-113	Annotation submitted by Leibniz Institute DSMZ	3500798	5	Complete Genome	11/26/2019 0:00	PacBio; Illumina	Leibniz Institute DSMZ	3581
GCA_009709115.1	*Piscirickettsia salmonis*	Psal-109	Annotation submitted by Leibniz Institute DSMZ	3512843	5	Complete Genome	11/26/2019 0:00	PacBio; Illumina	Leibniz Institute DSMZ	3588
GCA_009709295.1	*Piscirickettsia salmonis*	Psal-139	Annotation submitted by Leibniz Institute DSMZ	3512115	5	Complete Genome	11/26/2019 0:00	PacBio; Illumina	Leibniz Institute DSMZ	3601
GCA_009709095.1	*Piscirickettsia salmonis*	Psal-108	Annotation submitted by Leibniz Institute DSMZ	3531596	6	Complete Genome	11/26/2019 0:00	PacBio; Illumina	Leibniz Institute DSMZ	3620
GCA_009709175.1	*Piscirickettsia salmonis*	Psal-114	Annotation submitted by Leibniz Institute DSMZ	3512587	5	Complete Genome	11/26/2019 0:00	PacBio; Illumina	Leibniz Institute DSMZ	3595

GCA_010092545.1	*Piscirickettsia salmonis*	Ps-11091B	NCBI Prokaryotic Genome Annotation Pipeline (PGAP)	3516195	4	Complete Genome	2/1/2020 0:00	PacBio Sequel	CMM	3680
GCA_001932835.1	*Piscirickettsia salmonis*	PM58386B	NCBI Prokaryotic Genome Annotation Pipeline (PGAP)	3516383	5	Complete Genome	1/4/2017 0:00	PacBio; Illumina Hiseq	ADL Diagnostic Chile Ltda	3529
GCA_009709215.1	*Piscirickettsia salmonis*	Psal-118	Annotation submitted by Leibniz Institute DSMZ	3509947	5	Complete Genome	11/26/2019 0:00	PacBio; Illumina	Leibniz Institute DSMZ	3591
GCA_001932915.1	*Piscirickettsia salmonis*	PM31429B	NCBI Prokaryotic Genome Annotation Pipeline (PGAP)	3515768	5	Complete Genome	1/4/2017 0:00	PacBio; Illumina Hiseq	ADL Diagnostic Chile Ltda	3530
GCA_001932815.1	*Piscirickettsia salmonis*	PM49811B	NCBI Prokaryotic Genome Annotation Pipeline (PGAP)	3515614	5	Complete Genome	1/4/2017 0:00	PacBio; Illumina Hiseq	ADL Diagnostic Chile Ltda	3532
GCA_023008185.1	*Piscirickettsia salmonis*	Psal-104b	Annotation submitted by Leibniz Institute DSMZ	3482664	5	Complete Genome	4/15/2022 0:00	PacBio; Illumina	Leibniz Institute DSMZ	3556
GCA_009709135.1	*Piscirickettsia salmonis*	Psal-110	Annotation submitted by Leibniz Institute DSMZ	3516405	6	Complete Genome	11/26/2019 0:00	PacBio; Illumina	Leibniz Institute DSMZ	3598
GCA_009709015.1	*Piscirickettsia salmonis*	Psal-073	Annotation submitted by Leibniz Institute DSMZ	3547638	4	Complete Genome	11/26/2019 0:00	PacBio; Illumina	Leibniz Institute DSMZ	3596
GCA_001932935.1	*Piscirickettsia salmonis*	AY3864B	NCBI Prokaryotic Genome Annotation Pipeline (PGAP)	3515597	5	Complete Genome	1/4/2017 0:00	PacBio; Illumina HiSeq	ADL Diagnostic Chile Ltda	3538
GCA_001746795.1	*Piscirickettsia salmonis*	AY3800B	NCBI Prokaryotic Genome Annotation Pipeline (PGAP)	3515597	5	Complete Genome	9/26/2016 0:00	PacBio; Illumina Hiseq	ADL Diagnostic Chile Ltda	3539
GCA_001932855.1	*Piscirickettsia salmonis*	AY6297B	NCBI Prokaryotic Genome Annotation Pipeline (PGAP)	3515553	5	Complete Genome	1/4/2017 0:00	PacBio; Illumina Hiseq	ADL Diagnostic Chile Ltda	3541
GCA_001932875.1	*Piscirickettsia salmonis*	AY6532B	NCBI Prokaryotic Genome Annotation Pipeline (PGAP)	3489052	5	Complete Genome	1/4/2017 0:00	PacBio; Illumina Hiseq	ADL Diagnostic Chile Ltda	3516
GCA_009709435.1	*Piscirickettsia salmonis*	Psal-081	Annotation submitted by Leibniz Institute DSMZ	3508131	5	Complete Genome	11/26/2019 0:00	PacBio; Illumina	Leibniz Institute DSMZ	3580
GCA_001932895.1	*Piscirickettsia salmonis*	PM22180B	NCBI Prokaryotic Genome Annotation Pipeline (PGAP)	3509728	5	Complete Genome	1/4/2017 0:00	PacBio; Illumina Hiseq	ADL Diagnostic Chile Ltda	3520
GCA_000756415.3	*Piscirickettsia salmonis*	PM32597B1	Annotation submitted by ADL Diagnostic Chile Ltda	3509678	5	Complete Genome	8/31/2015 0:00	PacBio	ADL Diagnostic Chile Ltda	3644
GCA_003850185.1	*Piscirickettsia salmonis*	EM-90		3694262	9	Complete Genome	11/29/2018 0:00	PacBio	CMM	
GCA_014622645.1	*Piscirickettsia salmonis*	Ps12201A	NCBI Prokaryotic Genome Annotation Pipeline (PGAP)	3654436	13	Complete Genome	9/14/2020 0:00	Oxford Nanopore MinION	Instituto de Nutricion y Tecnologia de los Alimentos	4017

									- Universidad de Chile	
GCA_010092585.1	*Piscirickettsia salmonis*	Ps-8079A	NCBI Prokaryotic Genome Annotation Pipeline (PGAP)	3526646	5	Complete Genome	2/1/2020 0:00	PacBio Sequel	CMM	3787
GCA_001514395.1	*Piscirickettsia salmonis*	PSCGR01	NCBI Prokaryotic Genome Annotation Pipeline (PGAP)	3489744	5	Complete Genome	1/14/2016 0:00	Illumina; PacBio	University of Chile	3521
GCA_009708295.1	*Piscirickettsia salmonis*	Psal-005	Annotation submitted by Leibniz Institute DSMZ	3323804	2	Complete Genome	11/26/2019 0:00	PacBio; Illumina	Leibniz Institute DSMZ	3542
GCA_009709475.1	*Piscirickettsia salmonis*	Psal-104a	Annotation submitted by Leibniz Institute DSMZ	3452061	5	Complete Genome	11/26/2019 0:00	PacBio; Illumina	Leibniz Institute DSMZ	3718
GCA_009708795.1	*Piscirickettsia salmonis*	Psal-027	Annotation submitted by Leibniz Institute DSMZ	3450566	5	Complete Genome	11/26/2019 0:00	PacBio; Illumina	Leibniz Institute DSMZ	3715
GCA_009708835.1	*Piscirickettsia salmonis*	Psal-040	Annotation submitted by Leibniz Institute DSMZ	3484034	5	Complete Genome	11/26/2019 0:00	PacBio; Illumina	Leibniz Institute DSMZ	3729
GCA_009708915.1	*Piscirickettsia salmonis*	Psal-025	Annotation submitted by Leibniz Institute DSMZ	3523613	5	Complete Genome	11/26/2019 0:00	PacBio; Illumina	Leibniz Institute DSMZ	3781
GCA_009708975.1	*Piscirickettsia salmonis*	Psal-071	Annotation submitted by Leibniz Institute DSMZ	3452612	5	Complete Genome	11/26/2019 0:00	PacBio; Illumina	Leibniz Institute DSMZ	3693
GCA_009708735.1	*Piscirickettsia salmonis*	Psal-011	Annotation submitted by Leibniz Institute DSMZ	3424132	4	Complete Genome	11/26/2019 0:00	PacBio; Illumina	Leibniz Institute DSMZ	3671
GCA_009708695.1	*Piscirickettsia salmonis*	Psal-010a	Annotation submitted by Leibniz Institute DSMZ	3397484	5	Complete Genome	11/26/2019 0:00	PacBio; Illumina	Leibniz Institute DSMZ	3625
GCA_009708855.1	*Piscirickettsia salmonis*	Psal-041	Annotation submitted by Leibniz Institute DSMZ	3426373	5	Complete Genome	11/26/2019 0:00	PacBio; Illumina	Leibniz Institute DSMZ	3665
GCA_009708815.1	*Piscirickettsia salmonis*	Psal-028	Annotation submitted by Leibniz Institute DSMZ	3466089	5	Complete Genome	11/26/2019 0:00	PacBio; Illumina	Leibniz Institute DSMZ	3714
GCA_009708335.1	*Piscirickettsia salmonis*	Psal-006a	Annotation submitted by Leibniz Institute DSMZ	3465311	5	Complete Genome	11/26/2019 0:00	PacBio; Illumina	Leibniz Institute DSMZ	3722
GCA_009709455.1	*Piscirickettsia salmonis*	Psal-103	Annotation submitted by Leibniz Institute DSMZ	3444625	5	Complete Genome	11/26/2019 0:00	PacBio; Illumina	Leibniz Institute DSMZ	3686
GCA_009708255.1	*Piscirickettsia salmonis*	Psal-002	Annotation submitted by Leibniz Institute DSMZ	3432968	5	Complete Genome	11/26/2019 0:00	PacBio; Illumina	Leibniz Institute DSMZ	3694
GCA_009708875.1	*Piscirickettsia salmonis*	Psal-051	Annotation submitted by Leibniz Institute DSMZ	3454277	5	Complete Genome	11/26/2019 0:00	PacBio; Illumina	Leibniz Institute DSMZ	3700

GCA_009708775.1	*Piscirickettsia salmonis*	Psal-026	Annotation submitted by Leibniz Institute DSMZ	3392823	5	Complete Genome	11/26/2019 0:00	PacBio; Illumina	Leibniz Institute DSMZ	3632
GCA_001932795.1	*Piscirickettsia salmonis*	PM51819A	NCBI Prokaryotic Genome Annotation Pipeline (PGAP)	3429719	3	Complete Genome	1/4/2017 0:00	PacBio; Illumina Hiseq	ADL Diagnostic Chile Ltda	3621
GCA_009708995.1	*Piscirickettsia salmonis*	Psal-072	Annotation submitted by Leibniz Institute DSMZ	3714042	6	Complete Genome	11/26/2019 0:00	PacBio; Illumina	Leibniz Institute DSMZ	3956
GCA_009708935.1	*Piscirickettsia salmonis*	Psal-069	Annotation submitted by Leibniz Institute DSMZ	3375990	3	Complete Genome	11/26/2019 0:00	PacBio; Illumina	Leibniz Institute DSMZ	3631
GCA_009708275.1	*Piscirickettsia salmonis*	Psal-003	Annotation submitted by Leibniz Institute DSMZ	3380264	5	Complete Genome	11/26/2019 0:00	PacBio; Illumina	Leibniz Institute DSMZ	3617
GCA_009708315.1	*Piscirickettsia salmonis*	Psal-004	Annotation submitted by Leibniz Institute DSMZ	3378522	5	Complete Genome	11/26/2019 0:00	PacBio; Illumina	Leibniz Institute DSMZ	3607
GCA_009709415.1	*Piscirickettsia salmonis*	Psal-182	Annotation submitted by Leibniz Institute DSMZ	3390496	5	Complete Genome	11/26/2019 0:00	PacBio; Illumina	Leibniz Institute DSMZ	3615
GCA_009708235.1	*Piscirickettsia salmonis*	Psal-001	Annotation submitted by Leibniz Institute DSMZ	3406073	5	Complete Genome	11/26/2019 0:00	PacBio; Illumina	Leibniz Institute DSMZ	3669
GCA_009708895.1	*Piscirickettsia salmonis*	Psal-068	Annotation submitted by Leibniz Institute DSMZ	3403738	5	Complete Genome	11/26/2019 0:00	PacBio; Illumina	Leibniz Institute DSMZ	3658
GCA_000756435.3	*Piscirickettsia salmonis*	PM15972A1	Annotation submitted by ADL Diagnostic Chile Ltda	3245670	5	Complete Genome	8/21/2015 0:00	PacBio	ADL Diagnostic Chile Ltda	3365
GCA_009709395.1	*Piscirickettsia salmonis*	Psal-163	Annotation submitted by Leibniz Institute DSMZ	3302659	3	Complete Genome	11/26/2019 0:00	PacBio; Illumina	Leibniz Institute DSMZ	3449
GCA_009709355.1	*Piscirickettsia salmonis*	Psal-160	Annotation submitted by Leibniz Institute DSMZ	3336688	5	Complete Genome	11/26/2019 0:00	PacBio; Illumina	Leibniz Institute DSMZ	3471
GCA_009709335.1	*Piscirickettsia salmonis*	Psal-159	Annotation submitted by Leibniz Institute DSMZ	3325201	4	Complete Genome	11/26/2019 0:00	PacBio; Illumina	Leibniz Institute DSMZ	3477
GCA_009709315.1	*Piscirickettsia salmonis*	Psal-158	Annotation submitted by Leibniz Institute DSMZ	3327064	5	Complete Genome	11/26/2019 0:00	PacBio; Illumina	Leibniz Institute DSMZ	3476
GCA_009709075.1	*Piscirickettsia salmonis*	Psal-107	Annotation submitted by Leibniz Institute DSMZ	3364462	5	Complete Genome	11/26/2019 0:00	PacBio; Illumina	Leibniz Institute DSMZ	3524
GCA_009709375.1	*Piscirickettsia salmonis*	Psal-161	Annotation submitted by Leibniz Institute DSMZ	3396414	6	Complete Genome	11/26/2019 0:00	PacBio; Illumina	Leibniz Institute DSMZ	3574
GCA_001932775.1	*Piscirickettsia salmonis*	PM37984A	NCBI Prokaryotic Genome Annotation Pipeline (PGAP)	3285457	5	Complete Genome	1/4/2017 0:00	PacBio; Illumina Hiseq	ADL Diagnostic Chile Ltda	3394

GCA_001932735.1	*Piscirickettsia salmonis*	PM21567A	NCBI Prokaryotic Genome Annotation Pipeline (PGAP)	3349099	6	Complete Genome	1/4/2017 0:00	PacBio; Illumina Hiseq	ADL Diagnostic Chile Ltda	3469
GCA_001932595.1	*Piscirickettsia salmonis*	AY6492A	NCBI Prokaryotic Genome Annotation Pipeline (PGAP)	3354469	5	Complete Genome	1/4/2017 0:00	PacBio; Illumina Hiseq	ADL Diagnostic Chile Ltda	3464
GCA_001932955.1	*Piscirickettsia salmonis*	PM25344B	NCBI Prokaryotic Genome Annotation Pipeline (PGAP)	3511244	5	Complete Genome	1/4/2017 0:00	PacBio; Illumina Hiseq	ADL Diagnostic Chile Ltda	3640
GCA_001932755.1	*Piscirickettsia salmonis*	PM23019A	NCBI Prokaryotic Genome Annotation Pipeline (PGAP)	3308199	5	Complete Genome	1/4/2017 0:00	PacBio; Illumina Hiseq	ADL Diagnostic Chile Ltda	3460
